# Why the Fermi paradox may not be well explained by Wong and Bartlett’s theory of civilization collapse. A Comment on: 'Asymptotic burnout and homeostatic awakening: a possible solution to the Fermi paradox?' (2022) by Wong and Bartlett

**DOI:** 10.1098/rsif.2024.0140

**Published:** 2024-10-23

**Authors:** Chris J. Jackson, Christian Criado-Perez

**Affiliations:** ^1^ School of Management and Governance, University of New South Wales, Australia

**Keywords:** Fermi paradox, end of civilization, growth curves, crisis, singularity, exo-civilization

## Abstract

Wong and Bartlett explain the Fermi paradox by arguing that neither human nor extra-terrestrial civilizations can escape the time window singularity which, they claim, results from the way in which social characteristics of civilizations follow super-linear growth curves of cities. We question if data at the city level necessarily can lead to conclusions at the civilization level. More specifically, we suggest ways in which learnings from research, foresight, diversity and effective future government might act outside of their model to regulate super-linear growth curves of civilizations, and thus substantively increase the likelihood of civilizations progressing towards higher levels of the Kardashev scale. Moreover, we believe their claimed history of the collapse of terrestrial societies used to evidence their model is difficult to justify. Overall, we cast reasonable doubt on the ability of their proposed model to satisfactorily explain the Fermi paradox.

*J. R. Soc. Interface* 19: 1920220029 (Published online 04 May 2022). (http://doi.org/10.1098/rsif.2022.0029).

## 1. Introduction

Wong and Bartlett have recently developed a powerful scientific model to explain the Fermi paradox, which addresses why we have not yet come across alien civilizations [1]. We shall refer to this model as the Wong and Bartlett model or WBM. Based on the prior work of Bettencourt *et al*. [2], the WBM [1] argues that both human and extra-terrestrial civilizations across the universe will almost inevitably collapse or undergo homeostatic reawakening, which equates to achieving minimal growth. This is because social characteristics of civilizations characteristically follow super-linear growth curves such that their rate of growth becomes so fast that the time window to overcome singularities (or crises) eventually becomes too small to resolve. As a result of this ever-shortening time window, there is a bottleneck which causes almost all civilizations across the universe to collapse [1]. The aim of this article is to provide some reasons why the WBM [1] may not explain the Fermi paradox.

Wong and Bartlett [1] are keen to provoke discussion and are humble in their claims. In this spirit, we began to think about whether the two outcomes—civilization collapse or homeostatic awakening—are almost always the only outcomes. We will argue that they underestimate the chances of continued growth and technological advancement (albeit with times of reorganization) through the categories of the Kardashev scale [3]. We do not need to claim that continued civilization growth and technological advancement is a high probability outcome; we only need to claim that it is a non-negligible outcome to make our point that their model is limited in its application to explaining the Fermi paradox. It is important to note that we do not seek to disprove their ideas; instead, we aim to provide some arguments about why their conclusions might be premature. This viewpoint is especially worth discussing given that scholars are predicting the imminent downfall of human civilization because of climate change and other factors [4].

## 2. The levels of analysis issue in the Wong and Bartlett model

We argue that there are important factors at the civilization level, which are not included in the WBM [1] and which we theorize will generally act to promote continued civilization growth and technological advancement. Overall, our main argument coalesces into arguing that there is a levels of analysis fallacy (sometimes referred to as an ecological fallacy; cf. [5]) within the WBM [1], which occurs when observations and conclusions are made at one level (in this instance the evidence comes from individual super-linear growth curves associated with cities) and extended to represent conclusions at another level (outcomes of entire civilizations). The problem of this logic is that generalizing from one level of analysis to another is fraught with problems. While super-linear growth curves of individual social factors may be universal at a city level [2], we question if this leads to an almost inevitable bottleneck (collapse or homeostatic awakening) at the civilization level [1].

Some example levels of analysis errors from different disciplines in the literature will help illustrate the importance of the levels of analysis issue. While there is no relationship between individual-level dietary intake of fat and fatty acids with risk of breast cancer despite prior indications from international comparisons at the higher level—here higher level associations provide no guidance about lower individual-level associations [6]. In another study, aggregated data at the higher group level obscured important relationships between antibiotic prescribing and resistance at the lower person level [7]. In the context of physics and self-organization, the levels of analysis fallacy is illustrated when it is wrongfully assumed that chaotic behaviour at the local level implies chaotic behaviour at the global level; since in self-organizing systems, behaviour may be chaotic at the local level while stable and predictable at the global level [8,9].

We add detail to this perspective by arguing first that there is no evidence of terrestrial civilization collapse to date; and then we explore how some overlapping learning processes may act to undermine the conclusions drawn in the WBM [1] at the civilization level.

## 3. Historical evidence of collapse: collapse or reorganization?

Evidence in favour of the WBM [1] is presented through examples of human empire collapse across time. They illustrate civilization collapse with terrestrial examples such as Angkor, Babylon and Chichén-Itzá. We believe that this evidence is overstated; instead, similar to Eisenstadt [10], we advocate examples of collapse can often be interpreted as local collapses; but, at a broader level, they are also examples of reorganization. We thus advocate that these are not examples of terrestrial civilization collapse in its totality (i.e. at the higher level that could be variously defined as a the planetary, species or overall civilization level) but simply as lower level examples of collapse.

Let us look more deeply at the examples used by Wong and Bartlett [1]. The local collapses of the Angkor, Babylon and Chichén Itzá civilizations are often interpreted as resulting from a combination of environmental, social and political factors. In each case, there was likely a subsequent reorganization of their resources by successor states or societies. Angkor, the Khmer Empire’s capital, likely declined in the fifteenth century due to prolonged droughts, flooding and water management system stresses, compounded by invasions and internal strife. Its resources, including architectural knowledge, religion and urban infrastructure, were later integrated into regional developments such as under the Thai kingdoms [11]. Babylon fell in 539 BCE as a likely result of instability, economic decline and conquest by the Persian Empire under Cyrus the Great, who reorganized Babylon’s administrative and cultural resources to consolidate his rule [12]. Chichén-Itzá’s decline before and in the thirteenth century was likely caused by environmental problems such as droughts, overpopulation and resource depletion, together with internal conflicts. The resources and cultural heritage of Chichén-Itzá were absorbed and influenced subsequent Mayan city-states and later Mesoamerican civilizations [13].

The local collapse of the Western Ancient Roman Empire further illustrates our point. Its collapse resulted in the redistribution of its vast resources among various ‘barbarian’ kingdoms. Meanwhile, the Eastern Roman Empire flourished as it consolidated resources and power previously shared with the Western Empire. As a result of the local collapse of the Western Empire and the release from this burden, the Eastern Roman Empire enhanced its economic and military strength, as well as its trade, culture and administrative efficiency [14]. In all these examples and more generally, local civilization collapse is reorganization in which civilization more broadly builds persistence and resilience to achieve new strategic growth [15]. Others use similar words including regeneration [16], transformation [17] and revitalization [18]. More generally, reorganization fits partly within resilience theory which concerns how a system copes with disturbance [19].

We think it unlikely that human civilization as a whole has yet to experience collapse as proposed by the WBM [1]; but instead has merely experienced regional reorganizations that usually end up strengthening broader society in the longer term [16]. Overall, we believe that the examples of collapse proffered by the WBM [1] fail to provide any strong and unequivocal evidence of human civilization collapse and thus their attempt to explain the Fermi paradox is poorly substantiated by terrestrial history. If the evidence from the only known civilization is at the very least open to interpretation, we think there are dangers in using the history of terrestrial civilization to support the claim the WBM [1] can explain the Fermi paradox.

### 3.1. Learning from diversity and historical precedent within and between civilizations

Wong and Bartlett [1] are implicitly extrapolating data from city-level data [2] to making conclusions at the civilization level (and sometimes the species level). One factor omitted from the WBM [1] is that of diversity within and between cities and societies, which comprise civilizations, and between communicating civilizations. Life must have variations in its characteristics. We can be certain of this because all life must undergo general selection (natural selection is an example) and general selection requires variation in the population as well as selection and retention [20–23]. If there is a variation, then life will build varying cities and societies within a civilization, and between civilizations, to suit their characteristics as a way of reducing surprisal and minimizing individuals’ variational free energy [24–27]. Research already shows that growth curves are differentially affected by workforce skills, occupations and businesses as well as selective migration [28]. For example, growth curves of crimes vary within India (average *β* is 0.87 with a 95% CI of 0.81–0.93) and are different from those of other countries such as the United States and some Latin American countries in which the *β* is greater than 1 [29]. In general, cities within civilizations and different civilizations will be at different places on the growth curve as a result of their population, history and other characteristics including local ecology which will encourage some social characteristics and restrict others (e.g. a city by a sea or river might encourage tourism or trade). The basis of our argument here is that cities, societies and civilizations that are at an earlier point in the super-linear growth curve of a social characteristic have the opportunity to monitor and learn from those which are at more advanced levels of the super-linear growth curve. Various groups within such civilizations will consider how previous singularities across history were or were not avoided and mitigated. For example, there is a large literature, which studies how previous civilizations collapsed, and aims to draw lessons from such collapses [19]. Terrestrial governments commission research to learn lessons from history and other societies [30]. For example, the differing growth curves of COVID-19 across terrestrial societies and the different responses to it [31] led to significant overall learnings in terms international collaboration and individual national responses. Overall, the seriousness of the COVID-19 crisis was mitigated at least partially as a result of these learnings. Another example is how the Chernobyl nuclear disaster of 1986 has led to significant learnings across the world to reduce the chance of reoccurrence and mitigation of the effect of a reoccurrence [32].

In partial defence of the WBM [1], we recognize (i) that the super-linear growth curves of social characteristics of cities are reported against a background of variation which will occur within and between cities, (ii) that some governance structures in some cities aim to monitor and learn from what is happening in other cities and despite these factors (iii) that the super-linear growth curves of social variables at the city level still occur. Nonetheless, it is a substantial assumption to argue that cities have the same capacity to monitor and control the super-linear growth curves as regional and planetary civilizations. For example, responses to COVID-19 were usually operationalized at the country level [33] and operational guidelines for nuclear power stations are operationalized at a country level and by supranational groups [34].

One interesting further conclusion from our logic in this section, which we can draw from the WBM [1] is that human civilization as a whole benefits from the variation in governance resulting from the ways in which different countries conduct their affairs. The variability in government across nations promotes diversity and learning opportunities to monitor and mitigate super-linear growth curves to the benefit of humanity. We should also note that variation can also include some risks such as, for example, from rogue states.

### 3.2. Learnings from terrestrial governments of tomorrow may well be more effective than those of yesterday

We acknowledge that governments of the past have taken interest in controlling super-linear growth curves (e.g. ancient Roman empire’s attempt to control inflation during the third century). Nonetheless, effective control of unreasonable growth is often associated with contemporary terrestrial government policy [35–38]. For example, effective modern governments encourage and control economic growth using taxation, grants, training, policies on intellectual property and regulation of competition [39]. Optimal reform, regulation and public expenditure will hinge on accurate insights into future outcomes from effective sensing of trends and issues of importance, horizon scanning, planning and coordination [40]. This provides the opportunity to proactively avoid or respond to crises which will often mitigate both the occurrence and the seriousness of singularities associated with super-linear growth curves and contribute to sustainable growth. Thus, many modern governments have policies focused on renewable energy, environmental conservation, efficient resource utilization and responsible economic growth. One good specific example is the contemporary archiving of seeds in seed banks to mitigate the effects of possible civilization collapse [41]. If effective government of today is involved in at least partial regulation of super-linear growth, then we argue that the government of tomorrow may be superior in this capability. For example, we expect the on-going AI revolution to further develop the capacity of terrestrial governments to track super-linear growth curves and reduce the risk of collapse. As a result, the WBM [1] fails to include increasing government effectiveness in the future compared with the government effectiveness of the past because the data presented to support their model necessarily precede their paper. Again, we do not claim that all future governments will have superior capability compared with the past, but we do advocate that some will and that this important factor is excluded from their model.

Intriguingly, government capability to control collapse is itself likely to be a social variable with a super-linear growth curve. While such a possibility is in accord with the type of data [2] used in the WBM [1], it nonetheless acts at a higher level to monitor and control super-linear growth of other curves. The dominance of some super-linear growth curves over others is likely to be important in mitigating (or even promoting) collapse and are not included in the WBM [1].

### 3.3. Learnings from scholarly and other research on growth curves

Another important factor mitigating the certainty of a civilization-level singularity resulting from super-linear growth curves, and which is overlooked by Wong and Bartlett [1], is the effect of their own theoretical research (and other similar research). Their theoretical research highlights the dangers of super-linear growth and the resultant singularity and, just like other university research, it has the potential to impact government policy and thus outcomes. In general, scholarly and other research can provide foresight and forecasting on important social issues and thus help governments prepare for future scenarios [42]. Future thinking by researchers helps a government build resilient strategies and evaluate the success of existing strategies. Wong and Bartlett’s research [1], our own critique, and that of previous researchers [43] might impact policy directly or might need to be ‘translated’ by others, such as people in the media, into a more understandable format which is more easily absorbed by influential decision-makers including those in government and elites who may champion the cause [44,45].

A modern example highlights how academic research can affect policy and policy success on the international stage. Some scientists claim that the discovery of dendritic cells in 1961 can be considered the earliest pioneering milestone of mRNA-based vaccines. Thereafter, rigorous scientific research led to the discovery of critical knowledge in cell biology, which was crucial to the rapid deployment of the COVID-19 mRNA vaccines of contemporary times [45]. Such vaccines, and other academically driven research on pandemics, provided the bed-rock of government responses to the COVID-19 epidemic and the relatively successful mitigation of this singularity. Another compelling example is the large body of academic and other research focused on ways of controlling inflation which has led to partial success by western governments in preventing this social variable from developing super-linear growth curve characteristics [46].

More generally, there is reason to believe that effective government does heed the advice of academics and their research. As noted by Tyler [47, p. 7]: ‘For the most part, the UK Parliament has good systems for using evidence. Multidisciplinary units of politically impartial staff endeavour to answer all manner of questions for politicians, such us how planning regulations work or whether Wi-Fi radiation is safe. I spent five years directing one such unit, the Parliamentary Office of Science and Technology (POST), that proactively communicates academic research to politicians. Many legislatures globally have similar set-ups.’

Despite all these positive reasons why theoretical research is relevant to policymaking, we must acknowledge that there are many contemporary barriers to the uptake of academic research by policymakers [48]. For example, governments are often overwhelmed with information given the complexity of the world which makes it hard for them to identify the critical from the noise such that they can reduce surprise and organize suitable responses. We should also acknowledge how governments, often for political reasons, can ignore academic research as demonstrated by the current reluctance of many governments to drive change based on the strong evidence of climate research [49]. However, research also provides effective techniques for academics to overcome the barriers [50].

## 4. Summary

Wong and Bartlett [1] base their ideas on the reported super-linear growth curves of social variables of cities [2] and conclude that such growth curves will likely exist at the civilization level. Yet, Wong and Bartlett [1] provide little explanation of how these individual super-linear growth curves are associated with the overall super-linear growth curve associated with civilization collapse. Our critique focuses on the dangers of interpreting information from one level (i.e. individual super-linear growth curves of social variables of cities) in making conclusions at a different level (collapse of civilization). First, we noted that evidence of civilization collapse used by Wong and Barlett [1] is more at the local level than at the civilization level such that there is no compelling evidence to date of collapse. Second, we highlighted three civilization-level learning processes (diversity, developing government expertise and scholarly research) as being three influences outside of the WBM [1], which may act to reduce the efficacy of their conclusions. Nonetheless, we are keen to emphasize that our arguments are aimed more at adding to the debate about their model, as opposed to providing unequivocal evidence that undermines it.

As a result, we have some doubts as to whether the WBM [1] provides a good explanation for the Fermi paradox. We have more confidence that some civilizations—but not all civilizations—will have the capacity to control super-linear growth curves and mitigate the effects of singularities as they navigate their way up the Kardashev scale [3]. There are many other explanations of the Fermi paradox [51], which should be considered alongside the WBM [1]. Moreover, there are many other ideas of civilization collapse (e.g. [52–55]) which should be compared with the WBM [1].

Despite our misgivings, we believe that Wong and Bartlett’s [1] approach of modelling civilization-level growth curves using the same ideas as city-level growth curves has much merit and we do not wish to undermine the bulk of their ideas. In fact, we would like to stress that our comments stem from our enthusiasm and admiration for their ideas, and we seek to position our work as providing a possible note of caution on only the bigger claims regarding their model. The claims of super-linear growth of socially based variables of cities are well-founded [2] and data from urban scaling theory are generally supportive (e.g. [56,57]). More specifically, we agree that the *individual* social characteristics of civilizations can be *generally* understood in terms of super-linear growth curves—unless there is specific government or elite intervention following processes described in this paper; that an alternative to growth is homeostatic awakening; and that exo-civilizations might be expected to follow anthropomorphic data. Overall, we are very impressed with the WBM [1] and believe that it can form a strong basis for future work which can overcome the limitations highlighted in this article. We hope that our insights will be viewed as adding to the discussion and that they encourage more research at the civilization level into understanding the WBM [1].

## Data Availability

This article has no additional data.
